# The Spread of Non-Evidence-Based Health Claims on Social Media: The Case of #Mouthtape on Instagram, a Cross-Sectional Study

**DOI:** 10.3390/dj14070418

**Published:** 2026-07-08

**Authors:** Maria Eleonora Bizzoca, Christian Durante, Francisco José Vázquez Santos, Gennaro Musella, Lorenzo Lo Muzio, Eleonora Trecca, Alessandra Lucchese, Vito Carlo Alberto Caponio

**Affiliations:** 1 Department of Clinical and Experimental Medicine, University of Foggia, 71122 Foggia, Italy; mariaeleonora.bizzoca@unifg.it (M.E.B.); lorenzo.lomuzio@unifg.it (L.L.M.); 2 Department of Computer Science, University of Bari Aldo Moro, 70125 Bari, Italy; durante.christian17@gmail.com; 3 Department of Orthodontics, University of Santiago de Compostela, 15705 Santiago de Compostela, Spain; fvazquezsantos@gmail.com; 4 Unit of Otolaryngology, Department of Clinical and Experimental Medicine, University of Foggia, 71122 Foggia, Italy; eleonora.trecca@unifg.it; 5 Department of Life Sciences, Health and Health Professions, Link Campus University, Via del Casale di San Pio V 44, 00165 Rome, Italy; a.lucchese@unilink.it (A.L.); vca.caponio@unilink.it (V.C.A.C.)

**Keywords:** social media, misinformation, mouth-taping, Instagram, digital health literacy

## Abstract

**Background**: Social media platforms like Instagram have become major sources of health information, often amplifying unverified claims. Nocturnal mouth-taping is one such trend promoted as beneficial for sleep and overall health despite limited scientific evidence. **Methods**: In April 2025, 530 Instagram reels tagged #mouthtape were retrieved via the official API. After exclusions, 390 reels were analyzed. Two independent reviewers assessed each reel for relevance, stance on mouth-taping, presenter characteristics, engagement metrics, and content type. Spearman’s correlation, Mann–Whitney, Kruskal–Wallis, and Chi-Square tests were applied. **Results**: Overall, 94.4% of reels endorsed mouth-taping. Most content was produced by companies (55.4%), while only 11.6% was posted by medical professionals. Supportive posts had significantly higher shares than critical ones (mean 923 vs. 193; *p* = 0.015). Male presenters’ reels achieved more likes and shares, though female presenters had more followers. English-language posts generated greater engagement. Commercial and medical claims predominated, with limited representation of evidence-based information. **Conclusions**: Instagram content overwhelmingly promotes mouth-taping, largely driven by commercial actors. High engagement with supportive posts, regardless of scientific validity, underscores the role of algorithms in spreading non-evidence-based health claims.

## 1. Introduction

In the digital age, social media platforms have emerged as important information sources about health, frequently influencing both individual health decisions and public perceptions. Social media is becoming the main means of communication for millions of people globally, drastically changing the way information is produced, shared, and consumed. Platforms like Facebook, Instagram (IG), TikTok, and X (previously Twitter) allow users an easier and immediate access to the news than traditional media because of their interactive features and instantaneous information sharing [1]. As a result, information has become more democratic, and everyone can now voice their thoughts and take part in public discussions. Public opinion polarization, the unchecked spread of fake news, and information manipulation by interest groups, businesses, and governments are all becoming more-urgent problems. Gossipy or contentious content is frequently given priority by algorithms built to maximize engagement, which helps to create “information bubbles” that restrict exposure to different points of view [2].

One of the most significant problems of the digital age is the interconnectedness of the infodemic and misinformation [3]. In contrast to disinformation, which is the purposeful production and distribution of false material for manipulative ends, misinformation is the accidental spread of inaccurate or misleading information [4]. Contrarily, an infodemic is an abundance of information, frequently a combination of accurate, skewed, and wholly inaccurate data, that impairs people’s capacity to discern fact from fiction and hinders their ability to make well-informed decisions. These occurrences have especially serious repercussions during emergencies, like the COVID-19 epidemic, during which the spread of fabricated miracle treatments, conspiracy theories, and altered data jeopardized public health and healthcare institutions’ credibility [5]. Misinformation is even more difficult to control because of the speed at which it spreads on social media and messaging platforms. Algorithms that prioritize user engagement tend to favor viral content, which is often emotionally compelling or sensationalistic regardless of its veracity, and the growing sophistication of manipulation technologies, like deepfakes, makes it more difficult to distinguish between real and fake content [6,7].

IG stands out among these channels as a popular and highly visual platform on which health enthusiasts, influencers, and even medical professionals offer wellness tips. But there are still issues with the veracity and correctness of health information shared on social media, especially when there is insufficient or inaccurate scientific backing [8].

With billions of active users using it every day, IG is one of the most significant and popular social networks in the world [9]. Because of its visual-first strategy, which includes posts, stories, and reels, it is very popular within influencers, corporations, and individuals. By enabling users to interactively share their experiences, trends, and ideas, the platform functions as a potent instrument for social activism, personal expression, and business promotion. IG is becoming a major player in digital marketing and e-commerce.

One emerging health trend gaining traction on IG is the practice of (nocturnal) mouth-taping, the act of applying tape over the lips during sleep to promote nasal breathing. In order to promote nasal breathing, the procedure usually entails covering the middle part of the mouth with a piece of tape that extends from the philtrum to the inferior vermillion border [10].

Mouth-taping has been encouraged by a number of social media influencers who say it promotes nasal breathing and offers a number of advantages, including improved digestion, mood, and skin, more energy, a stronger immune system, and even a more defined jawline. Others claim it lessens cavities, gum disease, bad breath, and brain fog. The list of purported advantages has grown over the last several years as the fad has gained popularity; some people even claim that it has cured their sleep apnea and stopped them from snoring [10]. Mouth-taping has become a very popular wellness practice as a result of these increasing testimonials. Nevertheless, there are very few actual scientific studies on mouth-taping in this conversation [11].

In the context of social media research, it is important to distinguish between misinformation and content that is unsupported or not grounded in current scientific evidence. In this study, we do not aim to formally classify content as misinformation in a strict sense but rather to identify the prevalence of health-related claims that are not supported by available scientific data or that extend beyond current evidence.

Therefore, to ascertain whether IG is a trustworthy source of information about mouth-taping or whether it aids in the dissemination of misinformation, this study aimed to inspect the IG posts by looking at the amount of advertised content, type of providers and impact in terms of number of likes and shares.

## 2. Materials and Methods

This study was reported according to the Network, Object, Engine, Comparison, Observation guidelines for social media-based research [12]. The completed NOECO checklist is available as Supplementary Material (Supplementary Methods S2: Checklist for publication of social media–based research, the NOECO statement).

### 2.1. Sample Size Calculation

The target sample size was defined by estimating that 50% of the downloaded reels (total number available = ~17.000 posts present on IG (Meta Platforms, Inc., Menlo Park, CA, USA), accessed in April 2025) would be associated with medical content related to mouth-taping.

This proportion was adopted as a conservative estimate in the absence of prior data specifically addressing the prevalence of medical content on this topic on IG. The use of a 50% assumption is a standard approach in sample size calculation when the true proportion is unknown, as it maximizes variability and yields the largest required sample size, thereby ensuring adequate statistical power.

By setting a 95% confidence interval and a margin of error of 5%, the sample size was calculated to be 376 reels.

### 2.2. Reel Retrieval and Download

A script was developed for the automatic extraction of data relating to reel content published on IG using the official APIs, with subsequent saving of the data in an Excel file. The search was conducted through the most recent content method, which returns the media posted in the last 24 h containing the requested hashtag (#mouthtape).

To minimize the influence of algorithmic personalization, data collection was performed using the official Instagram Graph API, which retrieves publicly available content based on hashtag queries rather than user-specific feeds. Unlike front-end browsing, API-based retrieval is not influenced by individual user history, location, or prior interaction patterns, thereby enhancing reproducibility and reducing personalization bias.

Due to constraints of the Instagram Graph API, which allows querying one hashtag per request, a single hashtag (#mouthtape) was selected for data retrieval. This choice was made to ensure a standardized and reproducible data collection process. Among related hashtags, #mouthtape was chosen as it generated the highest volume of content and engagement at the time of data collection, thereby maximizing the relevance of the retrieved dataset.

The “most recent content” retrieval strategy was deliberately adopted to capture a real-time and less algorithmically filtered snapshot of the currently circulating content. Unlike the “top posts” option, which prioritizes content based on engagement metrics (e.g., likes, shares, and comments) and may therefore overrepresent highly popular or algorithmically amplified material, the “most recent” approach allows for a more neutral and chronological sampling of available posts. This strategy was chosen to better approximate the natural flow of content dissemination at the time of data collection and to reduce potential amplification bias.

On average, each API call returns about 50 reels, although the number may vary based on the flow of users posting content in the reporting period. Each item of media returned by the search is a media post from a public account. The detailed development environment is described in Supplementary Materials (Supplementary Methods S1: Instagram API–Based Data Collection Pipeline).

### 2.3. Content Evaluation

Two authors (M.E.B. and F.J.V.S.) independently revised the retrieved list of reels. In case of disagreement, consensus was achieved through discussion, with arbitration by a third senior reviewer when necessary (V.C.A.C.). Inter-rater agreement was assessed using Cohen’s kappa coefficient and showed excellent agreement between reviewers (κ = 0.88), supporting the reliability of the coding framework.

Specifically, they were asked to independently record for each reel: 1. whether relevant or not to mouth-taping; 2. number of comments, likes, and shares; 3. supporting or not the use of mouth-tape; 4. presenter’s sex; 5. original profile identity (laypeople; medical practitioner; companies; etc.); 6. number of followers of the publisher profile; 7. type of visual content (video, text, or both); 8. type of content (commercial; medical claims; people’s daily routine; etc.); and 9. language.

“Medical claims” were operationally defined as any statement suggesting health-related benefits, prevention, or treatment effects associated with mouth-taping (e.g., improvement in sleep quality, reduction in snoring, enhancement of systemic health, or prevention of oral diseases). To ensure consistency, reviewers followed predefined criteria and examples during the coding process.

Content categories were not mutually exclusive. Content was labeled as “commercial” when it primarily aimed to promote or sell a product, and as “medical claims” when it included health-related assertions, regardless of commercial intent. Therefore, a single reel could be classified under multiple categories if it simultaneously promoted a product and included health-related claims.

For the purpose of this study, content was not classified as “misinformation” per se. Instead, posts were categorized based on their stance (supportive vs. against) and the presence of health-related claims. Content was considered “unsupported” when health claims were presented without reference to scientific evidence or when they extended beyond currently available evidence in the literature. This approach was adopted to minimize subjective interpretation and to ensure a reproducible classification framework.

In addition, to evaluate the reliability and overall quality of the retrieved content, a validated quality appraisal tool was employed: the modified DISCERN (mDISCERN). The mDISCERN instrument assesses the reliability of health-related information across five domains, including the clarity of aims, transparency of information sources, balance and lack of bias, reference to supporting evidence, and acknowledgment of uncertainties. Each item has a yes/no judgement, scoring 1 for each yes, with a total sum of 5, as previously adopted [13,14].

Data collection complied with Instagram’s Terms of Service (https://help.instagram.com/termsofuse, accessed on 20 April 2025) and Meta Platform Policies (https://transparency.meta.com/it-it/policies/, https://developers.facebook.com/terms/) (both accessed on 20 April 2025). As in previous studies with a similar methodology, ethics approval was not required as no real patient data from our institutions were used. Favorable exception was given by the Ethics Committee for Research at University of Foggia with protocol number 0037677-VII/1. The results shown here cannot be traced back to individual posts [15,16,17]. All procedures were in full accordance with the Declaration of Helsinki.

We created a registered Meta Developer application, obtained approval for access to the “recent media” endpoint of the Instagram Graph API, and retrieved only publicly available video URLs (reels). These URLs were stored in an Excel file and subsequently provided to the team for analysis. No unauthorized scraping, data downloading, or collection of personal information was performed.

All the analyzed IG profiles were publicly available at the time of data collection, and only publicly accessible information was retrieved via the official API; thus, no private or personal data were accessed or processed.

### 2.4. Statistical Analysis

Shapiro–Wilk was employed to test for normal distribution of linear variables. As all the linear variables had a non-normal distribution (Shapiro–Wilk *p*-value < 0.001), correlation was explored by the Spearman rank test. To inspect differences in number of comments, likes, shares, and followers among groups, Mann–Whitney or Kruskal–Wallis tests were employed. Chi-Square test was performed to explore the association between categorical variables.

## 3. Results

An IG API query was completed, retrieving 530 reels. Of these, 26.4% (140) were not related to the mouth-tape; specifically, 57 were not available; and 19 reported duplicated content but in a different published reel. Twenty-five reels were related to nose strips and five to the general airway topic. Other reasons for exclusion were unrelated topics. Of 530 reels, 390 were related to mouth-taping (vs. 376 from sample size calculation) and were included in further analysis.

Spearman’s rank showed correlation among n. of followers, likes, comments and shares (Table 1).

Of the evaluated content, 368 reels (94.4%) were in support of the use of mouth-tape, with over 50% delivered by female presenters. The reels were published in 216 cases (55.4%) from companies, with very few profiles belonging to the medical field (11.6%). Notably, the observed proportion of content produced by healthcare professionals (11.6%) was substantially lower than the 50% estimate used for sample size calculation.

Only one university-hospital institution was found, and there were seven dentists. Commercial and medical contents were the most represented (respectively, 170 (43.6%) and 146 (37.4%)). Visual combined with text was the most common format (211, 54.1%). Almost all the reels were in English (334, 85.6%), followed by Spanish (25, 6.4%), Italian (11, 2.8%) and German content (5, 1.3%). The mean number of comments was 80, SD = 802, with 6834 likes with a maximum of 1,600,000 and 878 shares with a maximum of 230,000. Profiles reported an average number of 42,130 followers, with the biggest profile having 1,600,000 followers (Table 2).

Of the 15 reels against mouth-taping, six were shared by dentists or medical practitioners, five by laypeople, two by naturopaths or personal trainers, and two by companies. One provided educational content, while 14 reported medical claims in contrast with the current trend.

Shares were statistically significantly higher in the supportive vs. against reels, with a mean of 923, SD = 643 vs. 193, SD = 432 (Mann–Whitney *p*-value = 0.015). However, these differences should be interpreted with caution given the high variability observed across engagement metrics. Likes, comments, and n. of followers did not differ. The n. of comments was close to double for male presenters compared with females (mean 133 ± 1152 vs. 66 ± 476, Mann–Whitney *p*-value < 0.001); males had close to four times the number of likes as well (mean 13,001 ± 130,493 vs. 3213 ± 34,396, Mann–Whitney *p*-value = 0.003) and twenty times the shares (mean 2059 ± 19,033 vs. 124 ± 654, Mann–Whitney *p*-value = 0.013); however, female profile owners had almost double the followers (mean 32,314 ± 146,442 vs. 53,804 ± 138,844, Mann–Whitney *p*-value < 0.001).

Kruskal–Wallis showed statistically significant differences among the various profile identities. Companies received fewer comments than laypeople’s accounts, medical practitioners, and naturopath or personal trainers (Dunn-adjusted *p*-value Kruskal–Wallis < 0.001). Laypeople and naturopath or personal trainers also had higher numbers of followers and likes (Dunn-adjusted *p*-value Kruskal–Wallis < 0.001). Based on the content, medical claims and routines received more comments and likes than commercials (Dunn-adjusted *p*-value Kruskal–Wallis < 0.001). English content received statistically significantly more likes than other languages (Mann–Whitney *p*-value < 0.001).

The chi-Square test showed absence of a statistically significant difference between the sex of the presenter and support for using mouth-tape (*p*-value = 0.367), but companies and laypeople were more in support (*p*-value < 0.001). The chi-Square test also showed that companies engaged more male presenters and that there were more female presenters for content created by medical practitioners, laypeople, and naturopaths or personal trainers (*p*-value < 0.001). Medical practitioners almost exclusively delivered medical claims, as did neuropaths and personal trainers. Companies delivered more commercial content and also medical claims. Laypeople showed the use of mouth-tape in their daily routine, followed by medical claims (*p*-value < 0.001).

### Quality of Health Information

It is important to consider that mDISCERN was originally developed for the evaluation of structured health information materials, and its application to short-form audiovisual content such as IG reels may present inherent limitations. The highly condensed and informal nature of reels may not allow for comprehensive reporting of sources, uncertainties, or balanced perspectives.

Therefore, the low scores observed in this study may reflect not only poor information quality but also a partial mismatch between the assessment tool and the format being evaluated.

The mDISCERN tool was employed to judge the related quality of health information. Reliability was judged to be predominantly poor, and the information was presented in an unbiased and balanced way in only three cases (two of them presenting content against mouth-taping). The source of information was stated in only 10 cases, and six of these were health professionals. Areas of uncertainty were delivered in only five reels, of which two provided recommendations against the use of mouth-tape. In 160 reels the aim of the reel was clearly stated. Overall, the total mDISCERN score was 0 in 180 cases, while 3 and 4 were scored in only three and one cases, respectively, indicating the low quality available on IG. The total mDISCERN score did not correlate with the number of comments or with likes, shares, or followers.

## 4. Discussion

In the digital era, social media platforms, such as IG, have become central sources of health-related information, deeply influencing both individual health behaviors and public discourse. The immediacy of communication, combined with the potential for virality, allows health-related content, accurate or not, to reach millions of users within minutes. IG, with its visual-first interface and over 1.4 billion monthly active users, has become a particularly powerful medium for health promotion and wellness trends [9].

Within this media ecosystem, the practice of nocturnal mouth-taping—sealing the mouth during sleep to encourage nasal breathing—has gained widespread attention. Promoted by wellness influencers and lifestyle advocates, mouth-taping is credited with a wide range of alleged benefits, including improved sleep quality, energy, mood, digestion, clearer skin, better oral health, and even structural changes like a more defined jawline. Some testimonials go further, claiming resolution of snoring or even obstructive sleep apnea symptoms [10]. Despite the breadth of these claims, the current scientific literature provides only limited and often indirect support. The evidence base on mouth-taping itself is sparse, heterogeneous, and lacks large-scale, controlled trials. Most assertions stem from extrapolations about the benefits of nasal breathing and the negative effects of mouth breathing rather than from direct evaluations of mouth-taping as a clinical intervention [11].

Our findings revealed a pronounced imbalance in the representation of mouth-taping on IG. Over 90% of the analyzed content endorsed the practice, while only 5.6% was critical or neutral. Moreover, the vast majority of supportive posts were published by commercial entities (55.4%), while only 11.6% came from medical professionals or health institutions. Reels promoting mouth-taping also received significantly more shares than those that were critical, indicating a higher level of public engagement and visibility.

Interestingly, the proportion of healthcare-generated content was markedly lower than the conservative estimate (50%) used for sample size calculation. This discrepancy does not affect the validity of the sample size, as the initial assumption was intended to maximize variability in the absence of prior data. Rather, it represents a meaningful finding, highlighting the limited presence of healthcare professionals in the dissemination of mouth-taping-related content on IG.

Although some differences in engagement metrics reached statistical significance, the high variability observed suggests that these findings should be interpreted cautiously. The practical relevance of such differences remains uncertain, as engagement on social media platforms is often influenced by multiple factors, including outliers and algorithmic amplification.

This observation is consistent with previous findings on the “infodemic” phenomenon, in which sensational or emotionally appealing content tends to be amplified by social media algorithms, regardless of its factual accuracy [5]. Furthermore, the claim that mouth-taping can improve systemic health conditions, such as asthma, are largely unsupported or not consistently confirmed by the available clinical evidence. For instance, a crossover study conducted by Cooper et al. showed no benefit in asthma control from nighttime mouth-taping [18].

Algorithmic amplification plays a significant role in the popularity of these unsupported claims. As previously demonstrated during the Zika virus outbreak and the COVID-19 pandemic, misleading or incomplete content tends to outperform accurate information in terms of visibility and user interaction [5,19].

Compounding this problem is the fact that social media platforms are not neutral conduits of information. Their algorithmic structures favor content that elicits strong engagement, such as awe, fear, or enthusiasm, regardless of its medical validity. In our analysis, reels from non-medical sources, especially companies, not only dominated the conversation but also achieved greater reach, even when their claims lacked empirical support. This aligns with similar observations in the literature, in which commercial health content was shown to achieve higher engagement levels than evidence-based posts [8]. This contributes to “information bubbles” and complicates the dissemination of accurate health information. For example, on IG, reels supporting mouth-taping garnered statistically significantly higher shares than those opposing it, even when posted by companies rather than medical professionals. This may reflect algorithmic amplification mechanisms described in the prior literature.

Our finding that 37.4% of the IG content analyzed contained medical claims, including assertions that mouth-taping can alleviate snoring or cure sleep apnea, necessitates a comprehensive clinical discussion. Evidence from controlled clinical studies suggests that mouth-taping may transiently improve snoring and apnea–hypopnea indices in carefully selected mouth-breathers with mild obstructive sleep apnea under supervision [20]. However, these findings are limited to very small, monitored populations using hypoallergenic materials and cannot be extrapolated to the general public. While most claims circulating on social media are not supported by current evidence, it is important to distinguish between use in the general population and in selected clinical settings. Limited data from small, controlled studies suggest that mouth-taping may provide modest benefits in carefully selected individuals, such as mouth-breathers with mild obstructive sleep apnea under supervision. However, these findings are not generalizable and should not support widespread, unsupervised use. A recent systematic review [21] emphasized that, beyond this narrow subset, the practice offers minimal proven benefit and may carry potential risks, particularly in individuals with underlying conditions such as nasal obstruction or unrecognized sleep disorders. The poor reliability scores recorded by the mDISCERN evaluation underscore the potential patient safety risks associated with the mouth-taping trend. Most viral posts lacked reference to scientific evidence, disclosed no potential conflicts of interest, and presented anecdotal experiences as medical advice. Such low information quality, when coupled with persuasive visual content, amplifies non-evidence-based health claims and may influence self-administered practice without professional supervision. From a clinical standpoint, this type of content may contribute to delayed or inappropriate health-seeking behaviors. The contrast between these online narratives and the limited clinical evidence available is striking, as in this recent systematic review only 10 studies were included. While in some studies statistically significant improvements were observed in terms of the apnea–hypopnea index or snoring intensity, these do not necessarily correspond to clinical improvement or severity. Furthermore, due to small samples and diverse designs, some studies revealed statistically significant changes, while others did not, indicating uneven efficacy and low external validity. Only five of the ten included studies directly compared the apnea–hypopnea index between therapies. Two of these trials found no significant differences, and just three demonstrated a statistically significant reduction with intervention.

IG content also claimed prevention of cavities, reduction in morning bad breath and dry mouth, and included cosmetic benefits, such as promoting a sharper jawline. Currently, to our knowledge there is no study investigating these associations [10]. The current scientific literature regarding nocturnal mouth-taping is sparse, heterogeneous, and lacks consensus, yet it spotlights a few potential applications within airway management, predominantly centered on obstructive sleep apnea. For dental practitioners, otolaryngologists, and sleep medicine specialists, awareness of this trend is crucial. Dentists, in particular, often encounter patients exploring airway-focused therapies and are well positioned to provide evidence-based guidance discouraging unsupervised mouth-taping. These findings highlight the broader patient safety implications of social media-driven health behaviors and reinforce the importance of clinician engagement in digital health education.

This study presents several limitations that should be considered when interpreting the findings. As with most cross-sectional analyses of social media data, our sampling approach carries inherent constraints related to representativeness and temporal limitations.

Data were collected in April 2025, providing a snapshot of mouth-taping content at a single timepoint. Due to IG API restrictions, searches can only be performed using one hashtag per query; therefore, we selected #mouthtape, which yielded the highest number of posts and engagement metrics among related terms. Although this choice may have excluded content shared under alternative or compound hashtags, it ensured systematic retrieval within the platform’s operational constraints. Moreover, it is important to underscore the difficulty in obtaining a comprehensive and unbiased picture of health trends solely from social media content. For example, a previous study showed differences observed between Twitter and Sina Weibo, where Twitter had more false information published and shared, pointing to varying platform controls and user behaviors influencing information circulation [22].

The methodology used for content retrieval across platforms, such as hashtag-based searches, introduces its own biases. Time of posting, language, user location, and prior engagement history can all shape the visibility and virality of content, often skewing results in favor of highly engaging rather than medically accurate information. We tried to address this bias by relying on a standardized retrieval of the reels based on IG API access. However, this short-term snapshot remains methodologically consistent with previous social media infodemiology studies, providing valid insights into prevailing communication patterns at the time of analysis. Finally, as user-generated content cannot be externally verified, claims regarding health benefits or risks should be interpreted as communicative trends rather than factual medical statements.

Moreover, it is important to note that the present study does not formally adjudicate the accuracy of each claim but rather evaluates the extent to which widely disseminated content aligns with the current state of the scientific evidence.

The reliance on a single hashtag (#mouthtape) may have excluded related content indexed under alternative or compound hashtags (e.g., #mouthtaping, #nasalbreathing), potentially introducing selection bias.

In addition, the predominance of English-language content (85.6%) may introduce linguistic bias, potentially limiting the generalizability of the findings to non-English-speaking populations. Furthermore, only publicly available reels were included through the Instagram Graph API; therefore, private accounts, stories, and other non-accessible content were not captured, which may have influenced the representativeness of the dataset.

However, this approach ensured a standardized and reproducible retrieval strategy within the constraints of the IG API. Future studies could benefit from multi-hashtag or longitudinal approaches to provide a more comprehensive representation of the phenomenon.

Furthermore, data were collected over a 24 h period, representing a cross-sectional snapshot of content at a specific timepoint. Given the dynamic nature of social media platforms, content visibility and engagement may vary over time due to evolving trends and algorithmic prioritization. Therefore, the findings should be interpreted as reflecting prevailing communication patterns rather than stable or long-term dissemination trends. Despite these limitations, the use of a standardized API-based retrieval method enhances the internal consistency and reproducibility of the study.

Lastly, lack of evidence and guidelines on this specific topic make it difficult to critically evaluate the quality of the medical content.

## 5. Conclusions

This study highlights a significant mismatch between the popularity of nocturnal mouth-taping on IG and the current scientific evidence supporting its use. The overwhelming majority of reels analyzed endorsed mouth-taping, with most originating from commercial or non-medical profiles and only a small fraction representing healthcare professionals. Despite anecdotal claims of wide-ranging health benefits, the existing scientific literature remains limited, heterogeneous, and inconclusive, with no robust evidence to support most of the claims circulating online. These findings underscore the pressing need for well-designed clinical research to assess the safety and efficacy of mouth-taping, as well as for public health strategies aimed at improving digital health literacy and countering the dissemination of unsupported health claims on social media platforms.

This cross-sectional analysis of IG content related to mouth-taping revealed that supportive and promotional posts overwhelmingly outnumbered neutral or critical ones, with frequent inclusion of medical claims unsupported by scientific evidence. While the predominance of favorable narratives may reflect algorithmic amplification mechanisms described in the prior literature on wellness-oriented content, it also illustrates the ease with which visually driven unsupported health claims circulate. The study does not document adverse events or confirm causal links between exposure to such content and patient harm; rather, it identifies communication patterns that may contribute to shaping public perceptions and influencing self-administered health behaviors. These findings underscore the importance of vigilance among dental, medical, and public health professionals in monitoring social media trends and engaging in proactive, evidence-based education to mitigate the spread of unsupported health claims and promote informed decision-making.

From a practical standpoint, healthcare professionals should remain aware of emerging social media-driven health trends and address them during clinical encounters. Providing clear, evidence-based counseling, correcting misconceptions, and guiding patients toward appropriate care are essential steps in this context. Furthermore, strategic engagement in digital platforms may help improve the visibility of reliable information and counterbalance unsupported health claims.

Importantly, the present study does not assess user interpretation or behavioral outcomes, and therefore no direct conclusions can be drawn regarding the impact of this content on patient behavior or clinical safety.

## Figures and Tables

**Table 1:** Description of variables and correlations among engagement metrics.

Correlations						
			n. Comments	n. Likes	n. Shares	n. Followers
Spearman’s rho	**n. comments**	Correlation Coefficient	1.000	0.668 **	0.543 **	0.533 **
		Sig. (2-tailed)		0.000	0.000	0.000
	**n. likes**	Correlation Coefficient	0.668 **	1.000	0.585 **	0.679 **
		Sig. (2-tailed)	0.000		0.000	0.000
	**n. shares**	Correlation Coefficient	0.543 **	0.585 **	1.000	0.493 **
		Sig. (2-tailed)	0.000	0.000		0.000
	**n. followers**	Correlation Coefficient	0.533 **	0.679 **	0.493 **	1.000
		Sig. (2-tailed)	0.000	0.000	0.000	

** Correlation is significant at the 0.01 level (2-tailed).

**Table 2:** Distribution of content characteristics and descriptive statistics.

Variables		Count	Column N %	Mean	Standard Deviation	Minimum	Maximum
Supportive content	Against	15	3.8%				
	Supporting	368	94.4%				
	n.a.	7	1.8%				
Presenter sex	Female	206	52.8%				
	Male	154	39.5%				
	n.a.	30	7.7%				
Profile identity	Company	216	55.4%				
	Dental hygienist	1	0.3%				
	Dentist	7	1.8%				
	Laypeople	122	31.3%				
	Medical practitioner	12	3.1%				
	Naturopath practitioner	13	3.3%				
	Newspaper	7	1.8%				
	Personal trainer	11	2.8%				
	University hospital	1	0.3%				
Type of content	Commercial	170	43.6%				
	Educational	5	1.3%				
	Medical claims	146	37.4%				
	Routine	69	17.7%				
Type of media	Both	211	54.1%				
	Text	15	3.8%				
	Visual	164	42.1%				
Language	Arabic	1	0.3%				
	Bulgarian	1	0.3%				
	Croatian	1	0.3%				
	Czech	4	1.0%				
	Dutch	2	0.5%				
	English	334	85.6%				
	German	5	1.3%				
	Italian	11	2.8%				
	n.a.	1	0.3%				
	Polish	1	0.3%				
	Serbian	1	0.3%				
	Slovakian	1	0.3%				
	Spanish	25	6.4%				
	Turkish	2	0.5%				
n. comments				80.05	801.87	0.00	14,200.00
n. likes				6834.18	85,712.63	0.00	1,600,000.00
n. shares				878.69	11,984.36	0.00	230,000.00
n. followers				42,129.26	137,185.35	0.00	1,600,000.00

## Data Availability

The data that support the findings of this study are available from the corresponding author, Gennaro Musella, upon reasonable request. All relevant data are included within the article and its Supplementary Information files.

## Supplementary Materials

The following supporting information can be downloaded at: https://www.mdpi.com/article/10.3390/dj14070418/s1, Supplementary Methods S1: Instagram API-Based Data Collection Pipeline. Supplementary Methods S2: Checklist for publication of social media–based research, the NOECO statement.
