# Supplementary material for: The Spread of Non-Evidence-Based Health Claims on Social Media: The Case of #Mouthtape on Instagram, a Cross-Sectional Study
Source: Dent J (Basel). 2026 Jul 8;14(7):418. doi: 10.3390/dj14070418 (PMC13408839; doi:10.3390/dj14070418)
Supplement: Supplementary file 1 [file dentistry-14-00418-s001.zip › dentistry-4221377-Supplementary Methods S1.pdf]

Supplementary Methods S1: Instagram API-Based Data Collection Pipeline

Detailed description of the computational workflow used for Instagram data collection, including API authentication, hashtag-based retrieval, pagination, rate-limit management, quality control, and export of collected metadata.

Development environment:

- Python (3.13 version)
- Windows 11

Libraries used:

- requests, to make HTTP requests
- Pandas, for data management and manipulation
- OpenPyxl: for creating and editing .xlsx files

API setup procedure:

- Sign up on the Facebook for Developers platform with an IG Business account connected to Facebook
- Create an app on Meta Developer
- Apply for and obtain the following permits
    1. pages_show_list
    2. business_management
    3. instagram_basic
    4. instagram_manage_comments
    5. instagram_manage_insights

Subsequently, through the Facebook Developer Graph Explorer API tool, an Access Token containing the required permissions was generated, necessary for authentication to

the API. Access tokens generated through the Graph Explorer API are usually short-lived (1 hour). However, through the extension process, you can get tokens that are valid for up to 60 days.

Script workflow:

- Access Token Reading
  - read_access_token
    1. Input: The path to the .txt format file containing the Access Token
    2. Output: Access token to be used in API requests
    3. Procedure:
       a. The file is opened in read mode and the Access Token is read
- Retrieving Page ID and User ID
  - get_page_id
    1. Input: Access Token
    2. Output: ID of the Facebook page associated with the IG account
    3. Procedure:
       a. An API call is made to retrieve the ID of the Facebook Page associated with the IG Account
  - get_user_instagram_id,
    1. Input: Access Token and ID of the Facebook page associated with the IG account
    2. Output: ID of the IG business account ID
    3. Procedure:
       a. An API call is made to retrieve the IG business account ID [8]

- Hashtag ID retrieval
  - get_hashtag_id
    1. Input: Access token, hashtag name, and IG business account ID
    2. Output: ID of the hashtag mouthtape
    3. Procedure:
       a. An API call is made to retrieve the hashtag ID [9]
- Hashtag search by ID and start data collection
  - fetch_recent_media_hashtag
    1. Input: Access Token, Hashtag ID, User ID
    2. Output: A collection of reels with URLs, number of likes, and comments for each item
    3. Procedure:
       a. An API call is made to retrieve the recent content associated with the hashtag.
       b. Since the endpoint returns both photos, reels, and videos, URLs are filtered to only those that contain the string "/reel/"
       c. For each API call, the response includes a paging object containing the next URL. This URL is stored and used for the subsequent API request to retrieve the next batch of results, enabling efficient pagination and preventing the repeated retrieval of previously seen items.

          This loop continues until either no next page is provided by the API or a stopping condition (no new data returned for 5 consecutive requests) is met.

d. The extracted data (reels URL, number of comments, number of likes) are saved in a list called video_data. [10]

- Save collected data to Excel files (in .xlsx format)
  - save_video_urls_to_excel,
    - **i.** Input: The video_data list and the file name .xlsx
    - **ii.** Procedure:
      - **a.** If the file already exists, the data is updated without duplication
      - **b.** The data is organized into columns: Video URL, Number of Comments, Number of Likes
      - c. Links to reels are inserted as hyperlinks

Error handling and Rate Limit Management:

To ensure the stability and reliability of the script, error handling has been implemented

- Reading the file containing the Access Token

  The read_access_token function opens a .txt file containing the Access Token needed to authenticate to the API in read mode.

  Any errors such as file not found (FileNotFoundError) or permissions issues are handled by interrupting the process and notifying the user

- Facebook and IG API calls

  All functions that interact with APIs (get_page_id, get_user_instagram_id, get_hashtag_id, fetch_recent_media_hashtag) implement checks on the HTTP status code of the response:

  1. If the HTTP response is other than 200 (success), an error message is printed containing the status code and the contents of the response

2. In the event of 429 Too Many Requests response (exceeding the maximum quota of calls allowed), the script starts a sleep of 3600 seconds before retrying the request, avoiding premature termination of the process
3. Incorrect responses are analyzed, trying to extract and display any error messages returned by the server

- API Call Limit Management when the fetch_recent_media_hashtag function is executed, the X-RateLimit-Remaining header is monitored:
  1. If the number of remaining calls falls below a critical threshold (200 remaining requests), an automatic pause of 3600 seconds is triggered to avoid incurring errors of exceeding the limits, this control is due to the limits imposed by IG for calls
- Data retrieval management
  Within the fetch_recent_media_hashtag function, in case no new data is returned for 5 consecutive calls, the collection process is automatically stopped, preventing infinite loops or unnecessary requests
- Writing to Excel Files
  In the save_video_urls_to_excel function:
  1. If the target file is already there, the list of videos already saved is checked to avoid duplication
  2. If the destination folder is missing, it is created dynamically.
  3. Potential errors related to writing to files (e.g. open file or insufficient writing permissions) are handled by preventing the program from crashing unexpectedly

Limitations:

During the development and use of the script, some operational limitations emerged:

1. The volume of results is highly dependent on organic traffic on IG in the last 24 hours
2. The API returns a mixture of content types (photos, videos, reels); reels had to be manually filtered
3. "Top Media" content cannot be accessed reliably, as selection criteria are not documented, and reels are not returned in this mode
