# Supplementary material for: The Spread of Non-Evidence-Based Health Claims on Social Media: The Case of #Mouthtape on Instagram, a Cross-Sectional Study
Source: Dent J (Basel). 2026 Jul 8;14(7):418. doi: 10.3390/dj14070418 (PMC13408839; doi:10.3390/dj14070418)
Supplement: Supplementary file 1 [file dentistry-14-00418-s001.zip › dentistry-4221377-Supplementary Methods S2.pdf]

Supplementary Methods S2: Checklist for publication of social media–based research, the NOECO statement.

Section/topic	No	Item	Page reported
Title	1	Identify manuscript as an analysis of social media data using specific analytical tools.	1
Abstract			
Summary	2	Report the background; objectives: including the data source and time frame; methods: including analytical engine to extract the data as well as data management tools; results: description of raw data, description of post-analysis data and limitations; conclusions: key findings.	1
Introduction			
Rationale	3	Describe what is already known about the topic and the rationale for the data extraction and analysis.	2–3
Objectives	4	Provide an explicit statement of questions being addressed with reference to defining the network and what is being evaluated, compared, and observed (**NOECO – Network, Object, Engine, Comparison, Observation**).<br>**Network**: Defined as the digital platform where users (nodes) share resources such as data. Examples include Facebook and Twitter.<br>**Object**: Defined as the component of the network to be studied. It can consist of the users of the network, a particular set of data shared among them of the structures created by these interactions. Examples include tweets around a hashtag, sharing of a particular content or sentiment analysis on a particular population.<br>**Engine**: Defined as a networks analysis tool used to measure the objects. This is typically a proprietary software able to mine and analyze large amounts of	3

Section/topic	No	Item	Page reported
		data. Examples include NodeXL and Gephi.<br>**Comparison**: Defined as the comparison against which the measure is made, similar to comparison between intervention and control groups. Examples include the number of interactions between users in Twitter compared to the same users in Facebook.<br>**Observation**: Defined as the actual observation hypothesized for the study. Examples include healthcare users of Twitter that are clustered around few sources.	
Methods			
Protocol	5	Indicate whether a protocol (i.e., a pre-defined method to undertake the evaluation of the social media data) exists, if it was created prior to the data extraction and analysis, and where it can be accessed (e.g. permalink at website).	Not reported
Data source	6	Describe the data source in terms of platform and type of data (e.g., raw data, filtered by the researchers, or managed by platform automatically).	3–5
Data appropriateness	7	Describe theoretical frameworks, characteristics of the data, inferences about data, and inferences about users. (e.g., does the data that is suggested to be used have internal validity for the question that is being asked.)	3–5, 10–11
Data inclusion	8	Describe data to be included and search strategy to be used and rationale.	3–5
Data exclusion	9	Describe data to be excluded, nodes or uses to be excluded, (e.g., suspected spam [automatic commercial offerings] or bots [automatic nodes designed to influence networks]), and data arguments to be excluded and rationale.	5

Section/topic	No	Item	Page reported
Data extraction	10	Describe data extraction engine to be used, program interface version if available, output format, and corruption data percentage. Describe how data was filtered.	3–5
Data analysis	11	Describe analytical tool used, cite pertinent papers describing methods of the tool, and describe the output format of the data. If analysis is performed by the data extraction engine itself, the underpinning (e.g., network centrality calculation – who/what are the most important people or nodes in a network) methodology should be described.	5
Synthesis of results	12	Describe the statistical analysis tool (e.g., univariate analysis), specifically if using large datasets statistical tools (e.g., eigenvectors).	5
Results			
Data selection	13	Provide platform, dates, and magnitude of the data points and search strategy.	5–9
Data corruption	14	Provide magnitude of data corruption, contamination (spam bots), unobtainable or missing data. Describe source of corruption/bias.	5
Data quality	15	Describe whether the data quality is appropriate in terms of size, corruption and ability to make appropriate inferences. Describe whether the Objects and Engine (from NOECO) were appropriate.	8–11
Analysis	16	Describe how the data analysis supports or disproves the original question. Describe whether end points or surrogate markers were met. Describe the Comparison and Outcomes from the NOECO question.	6–11
Discussion			

**Section/topic**	**No**	**Item**	**Page reported**
Summary	17	Describe the main findings in the dataset, i.e., how they do (or do not) answer the NOECO data question.	9–13
Limitations	18	Describe data source, set, and analysis limitations.	11–12
Conclusions	19	Provide a general interpretation of the data question after the data analysis.	13
Disclosures	20	Describe sources of funding, support, and conflict of interest, particularly regarding proprietary data extraction and analysis tools.	13
